# Prediction of Concrete Compressive Strength Based on ISSA-BPNN-AdaBoost

**DOI:** 10.3390/ma17235727

**Published:** 2024-11-22

**Authors:** Ping Li, Zichen Zhang, Jiming Gu

**Affiliations:** School of Management Science and Engineering, Anhui University of Technology, Ma’anshan 243002, China; 20150009@ahut.edu.cn (P.L.); zichenzhang12138@163.com (Z.Z.)

**Keywords:** back propagation neural network, sparrow search algorithm, concrete, compressive strength prediction, adaptive boosting

## Abstract

Strength testing of concrete mainly relies on physical experiments, which are not only time-consuming but also costly. To solve this problem, machine learning has proven to be a promising technological tool in concrete strength prediction. In order to improve the accuracy of the model in predicting the compressive strength of concrete, this paper chooses to optimize the base learner of the ensemble learning model. The position update formula in the search phase of the sparrow search algorithm (SSA) is improved, and piecewise chaotic mapping and adaptive t-distribution variation are added, which enhances the diversity of the population and improves the algorithm’s global search and convergence abilities. Subsequently, the effectiveness of the improvement strategy was demonstrated by comparing improved sparrow search algorithm (ISSA) with some commonly used intelligent optimization algorithms on 10 test functions. A back propagation neural network (BPNN) optimized with ISSA was used as the base learner, and the adaptive boosting (AdaBoost) algorithm was used to train and integrate multiple base learners, thus establishing an adaptive boosting algorithm based on back propagation neural network improved by the improved sparrow search algorithm (ISSA-BPNN-AdaBoost) concrete compressive strength prediction model. Then comparison experiments were conducted with other ensemble models and single models on two strength prediction datasets. The experimental results show that the ISSA-BPNN-AdaBoost model exhibits excellent results on both datasets and can accurately perform the prediction of concrete compressive strength, demonstrating the superiority of ensemble learning in predicting concrete compressive strength.

## 1. Introduction

Concrete is a very commonly used civil engineering material, and its strength directly determines the safety and durability of the structure. Traditionally, strength testing of concrete relies on physical experiments, which are time-consuming and costly due to the highly discrete nature of concrete experiments. Machine learning, as a powerful data analysis tool, has shown great potential in predicting concrete strength, which can be used as a reference for practical engineering. Machine learning models can assist engineers in making more accurate decisions during the design phase by analyzing historical data and experimental results to predict the performance of concrete. This usually requires the model to be trained and validated and involves extensive data collection and analysis. Moreover, machine learning can also be used in conjunction with multi-objective optimization algorithms to develop concrete mixtures that meet specific project requirements [1], again providing valuable guidance during the design process.

Artificial neural networks (ANN) are now widely used in concrete strength prediction. They consist of multiple neurons, each of which receives input signals and generates output signals through computation. Many researchers [2,3,4] have selected back propagation neural network (BPNN) by taking the material parameters, curing conditions, age, and specimen size of the constituent concrete as inputs to the prediction model and have developed a model for predicting the compressive strength of concrete using a database of actual concrete mixture ratios. The performance evaluation results show that the BPNN on their datasets has good predictive ability with a goodness of fit greater than 0.9, which outperforms traditional regression models in terms of accuracy. Some researchers have also predicted the 28-day compressive strength of ash silica fume self-compacting concrete [5] and lightweight foam concrete [6] using support vector machine (SVM) and concluded that the control parameters of SVM are more concise and also achieved better prediction results on their datasets. Other researchers have also combined optimization algorithms with some models to further optimize the hyperparameters of the models, thus improving the accuracy of the prediction results. Huang et al. [7] combined the simulated annealing algorithm (SA) with the particle swarm optimization algorithm (PSO) to establish an ASAPSO-ANN model for predicting the compressive strength of rubber concrete and compared it with the ANN and PSO-ANN models, and the results showed that the accuracy of predicting the strength of rubber concrete was improved. Li et al. [8] selects three machine learning methods, random forest (RF), k-nearest neighbors (KNN), and SVM, to predict the compressive strength of ultra-high performance concrete (UHPC) and also optimizes the predictive model hyper-parameters by using three meta-heuristic optimization algorithms, PSO, beetle antenna search (BAS), and serpentine optimization (SO), and the results show that random forests based on serpentine optimization have the highest predictive performance.

In addition to the above improvements, in recent years, researchers have proposed ensemble learning as a machine learning paradigm for improving model performance. This paradigm aggregates various base learning models into an ensemble that leverages the strengths of each component, aiming to decrease the generalization error and enhance the predictive accuracy of the overall model. The current ensemble learning methods are boosting, bagging, and stacking [9]. Ahmad et al. [10] used the bagging algorithm to predict the compressive strength of concrete, and the results showed that the ensemble learning model gave more accurate results as compared to DT and gene expression programming (GEP). Li et al. [11] has trained the established dataset of compressive and tensile strength of high-strength concrete using four ensemble learning models, adaptive boosting (AdaBoost), gradient boosting decision tree (GBDT), extreme gradient boosting (XGBoost), and RF, to obtain the optimal dataset splitting ratio as well as the sensitivity of the input variables, and the best predictive performance was obtained for the GBDT model. To predict the compressive and flexural strengths of mixtures containing recycled concrete aggregate (RAC), tree-based and augmented integrated machine learning models were developed by Martini et al. [12]. These models predict the compressive and flexural strengths of mixtures containing recycled concrete aggregates more accurately based on the constituent materials of RAC. Among the machine learning models it considered, the XGBoost model demonstrated the highest prediction performance. Li et al. [13] developed a stacking ensemble learning-based compressive strength prediction model for rice husk ash (RHA) concrete, using the ensemble learning model for the first layer of the stacked model and a linear regression model for the second layer of the stacked model, and verified the reasonableness of the base learner selected in the stacked model and the superiority of the stacked integration strategy. The abovementioned researchers obtained good prediction results using ensemble learning models. However, improving the performance of basic learners is also a good way to enhance overall performance.

Therefore, in order to improve the accuracy of the model in predicting the compressive strength of concrete, this paper chooses to optimize the base learner of the ensemble learning model. The position update formula in the search phase of the sparrow search algorithm (SSA) is improved, and piecewise chaotic mapping and adaptive t-distribution variation are added, which enhances the diversity of the population and improves the algorithm’s global search and convergence abilities. Subsequently, the effectiveness of the improvement strategy was demonstrated by comparing the improved sparrow search algorithm (ISSA) with some commonly used intelligent optimization algorithms on 10 test functions. A BPNN optimized with ISSA was used as the base learner, and the AdaBoost algorithm was used to train and integrate multiple base learners, thus establishing an adaptive boosting algorithm based on the back propagation neural network improved by the improved sparrow search algorithm (ISSA-BPNN-AdaBoost) concrete compressive strength prediction model. The purpose of optimizing the BPNN using ISSA is to find the optimal weights and thresholds for the network through a global search strategy to avoid falling into local optimal solutions in order to improve the performance and generalization of the network. The ISSA-BPNN can obtain better prediction performance than normal BPNN, and it can improve the performance of the integrated learner combined with AdaBoost. Then simulation experiments were conducted with other ensemble models and single models on two strength prediction datasets. The experimental results show that the ISSA-BPNN-AdaBoost model exhibits excellent results on both datasets and can accurately perform the prediction of concrete compressive strength. It can be used to provide design references in real projects [14].

## 2. Optimization Algorithm and Improvements

### 2.1. Sparrow Search Algorithm

The SSA [15] is a heuristic optimization algorithm that was proposed in 2019. The basic principle of the SSA is to divide a sparrow population into discoverers who find food and joiners who pursue the discoverer and at the same time introduce an alert detection mechanism, which selects a certain proportion of individuals to become scouts for detection and warning. In this process, the identities of the discoverer and joiner are not static, but their proportions in the sparrow population are fixed [16].

The discoverer with better fitness values shows stronger food-seeking ability, prioritizes food acquisition during the search process, and has a larger foraging search range than the joiner. During each iteration, the position of the discoverer is updated with the following formula:

(1) $$X_{i,j}^{t+1}=\left\{\begin{array}{c} X_{i,j}^{t}\cdot \exp\left(\frac{-i}{\alpha \cdot {iter}_{max}}\right)\;{\;R}_{2}<ST\;\\ X_{i,j}^{t}+QL\;{\;R}_{2}\geq ST \end{array}\right.$$

where t represents the current iteration number. ${iter}_{max}$ presents the maximum number of iterations. $X_{i,\;j}$ represents the position information of the ith sparrow in the jth dimension. $\alpha \left(\alpha \in \left(0,1\right)\right)$ is a random number. Q is a random number that obeys a normal distribution. L represents a 1×d matrix, where each element in the matrix is 1. When $R_{2}<ST$, there are no predators around the foraging environment at this time, and the finder can perform extensive search operations. When ${\;R}_{2}\geq ST$, a predator has been detected, and the sparrow population receives an alert, at which point all sparrows need to quickly fly to other safe places [15].

The joiner follows the finder, following foraging or competing for food, and the position of the joiner is updated with the following formula:

(2) $$X_{i,j}^{t+1}=\left\{\begin{array}{c} Q\cdot \exp\left(\frac{\left(X_{worst}^{t}-X_{i,j}^{t}\right)}{i^{2}}\right)\;i>\frac{n}{2}\\ X_{best}^{t+1}+\left|X_{i,j}^{t}-X_{best}^{t+1}\right|A^{+}\cdot L\;otherwise \end{array}\;\right.$$

where $X_{best}^{t+1}$ represents the optimal position for the iteration t+1. $X_{worst}^{t}$ represents the worst position for the iteration. A is a 1×d matrix where each element is randomly assigned a value of 1 or −1, and $A^{+}=A^{T}{\left(AA^{T}\right)}^{-1}$. When $i>\frac{n}{2}$, the ith joiner is not getting food and is in a very hungry state and therefore needs to relocate in order to forage for food [15].

When aware of the danger, the scout will alert, and the sparrow population will engage in antipredator behavior with the following equation:

(3) $$X_{i,j}^{t+1}=\left\{\begin{array}{c} X_{best}^{t}+\beta \cdot \left|X_{i,j}^{t}-X_{best}^{t}\right|\;{\;f}_{i}\neq f_{b}\;\\ X_{i,j}^{t}+K\cdot \left(\frac{X_{i,j}^{t}-X_{worst}^{t}}{\left(f_{i}-f_{w}\right)+\varepsilon }\right){\;f}_{i}=f_{b} \end{array}\right.$$

β is a step control parameter, which is a random number that obeys a normal distribution with mean 0 and variance 1. $K\left(K\in \left[-1,1\right]\right)$ is a random number. $f_{i}$ represents the current fitness value of an individual sparrow. ε is a very small constant to avoid zero in the denominator. When $f_{i}\neq f_{b}$, it means that the sparrow is at the edge of the population and is vulnerable to predators. When $f_{i}=f_{b}$, it means that the sparrow in the middle of the population is aware of the danger and needs to move closer to the other sparrows in order to minimize the risk of being preyed upon [15].

The SSA has a better ability to find the optimum, the overall convergence speed is faster, and the algorithm has fewer parameters [17]. However, it also has some shortcomings. The quality of the initial population of the SSA is lower, which affects the efficiency of the algorithm to a certain extent. From the updating formula of joiner position in the SSA, it can be seen that when $R_{2}<ST$, each dimension of the population is getting smaller, and the convergence will be worse when the extreme value of the objective function is not at the origin. In addition, when the discoverer is located in the local optimum, the joiner will follow the aggregation to the local optimum position, resulting in a poor ability to jump out of the local optimum. Since the number of individuals of the discoverer and the joiner is constant in the SSA, this leads to the process of the algorithm not being able to affect the iteration, especially when the algorithm enters into the later iteration, and the local optimum convergence stagnation phenomenon will be further deteriorated.

### 2.2. Improvement of the SSA

In this paper, we consider the following three strategies to optimize the SSA into the improved sparrow search algorithm (ISSA).

#### 2.2.1. Adding Piecewise Chaotic Mapping

It has been proven by many experiments [18,19,20,21] that the use of chaotic sequences for population initialization can have an impact on the whole process of the algorithm, and the fitness value of the random numbers generated by using chaotic mapping has been significantly improved. It is easier to search for the globally optimal solution, and it is effective to increase the diversity and randomness of the algorithm, especially when there are many local solutions in the search space. After testing many kinds of chaotic mapping, adding piecewise chaotic mapping can effectively increase the diversity of sparrow initialization and optimize the performance of the algorithm. The formula of piecewise chaotic mapping is as follows:

(4) $$X_{t+1}=\left\{\begin{array}{c} X_{t}/p\;0\leq X_{t}<p\\ X_{t}-p/0.5-p\;p\leq X_{t}<0.5\\ 1-p-X_{t}/0.5-p\;0.5\leq X_{t}<1-p\\ 1-X_{t}/p\;1-p\leq X_{t}<1 \end{array}\right.$$

where $p\left(p\in \left[0,1\right]\right)$ is a random number.

#### 2.2.2. Improving the Discoverer Strategy

The northern goshawk optimization algorithm (NGO) was proposed in 2022 [22]. This algorithm simulates the behavior of a northern goshawk during predation, including the phases of prey recognition and attack (global search) and pursuit and escape (local search). Since the selection of prey in the search space is randomized, which is equivalent to a global search of the space, this phase increases the exploration capacity of the NGO [23]. In order to improve the adequacy of the discoverer’s search in the solution space in the SSA, the discoverer’s position update formula when $R_{2}<ST$ is replaced with the NGO’s position update formula for the exploration phase. The position update formula for the exploration phase of the NGO is as follows:

(5) $$P_{i}=X_{k},\;i=1,2,\cdots ,\;N,\;k=1,2,\cdots ,i-1,i+1,\cdots ,N$$

(6) $$X_{i,j}^{new,P1}=\left\{\begin{array}{c} X_{i,j}+r\left(p_{i,j}-IX_{x,y}\right)\;Fp_{i}<F_{i}\\ X_{i,j}+r\left(X_{i,j}-p_{x,y}\right)\;Fp_{i}\geq F_{i} \end{array}\right.$$

where $P_{i}$ represents the position of the ith goshawk’s prey. $Fp_{i}$ represents the value of the objective function for the position of the ith goshawk’s prey. $F_{i}$ represents the value of the objective function that is solved for. k is a random number in the range of $\left[1,N\right]$. r is a random number in the range of $\left[0,1\right]$. I is a random number of 1 or 2.

#### 2.2.3. Adding Adaptive t-Distribution Variation

In heuristic algorithms, the introduction of variation is beneficial to improve the ability of the algorithm to jump out of the local optimum, because the variation operator enables the algorithm to have a certain local stochastic search ability, allowing it to accelerate the convergence to the optimal solution in the later stages of the solution while maintaining the diversity of the solutions [24,25]. In this paper, adaptive t-distribution variation is introduced to improve the search strategy of the algorithm, and t-distribution perturbation variation is performed with a certain probability in the follower stage of SSA, which enables the algorithm to explore the search space efficiently at the early stage of the evolution and develop the local optimal solution more accurately at the later stage of the evolution. The specific modes of positional variation are as follows:

(7) $$X_{new}^{i}=X_{best}^{j}+t\left(C_{iter}\right)\cdot X_{best}^{j}$$

where $X_{new}^{i}$ represents the position of the optimal solution in the jth dimension after the variational perturbation. $X_{best}^{j}$ is the position of the optimal solution in the jth dimension before the variational perturbation. The number of iterations is used as a parameter for the degrees of freedom of the t-distribution, where $t\left(C_{iter}\right)$ carries out the variant.

### 2.3. Optimization Algorithm Performance Testing

In order to test the performance of the ISSA, the ISSA is compared with dung beetle optimizer (DBO) [26], NGO, SSA, and gray wolf optimizer (GWO) [27] in experiments on 10 international common test functions. These functions usually have known optimal solutions or near-optimal solutions and can be used to test the search ability, convergence speed, and accuracy of the optimization algorithms.

**Figure 1:**
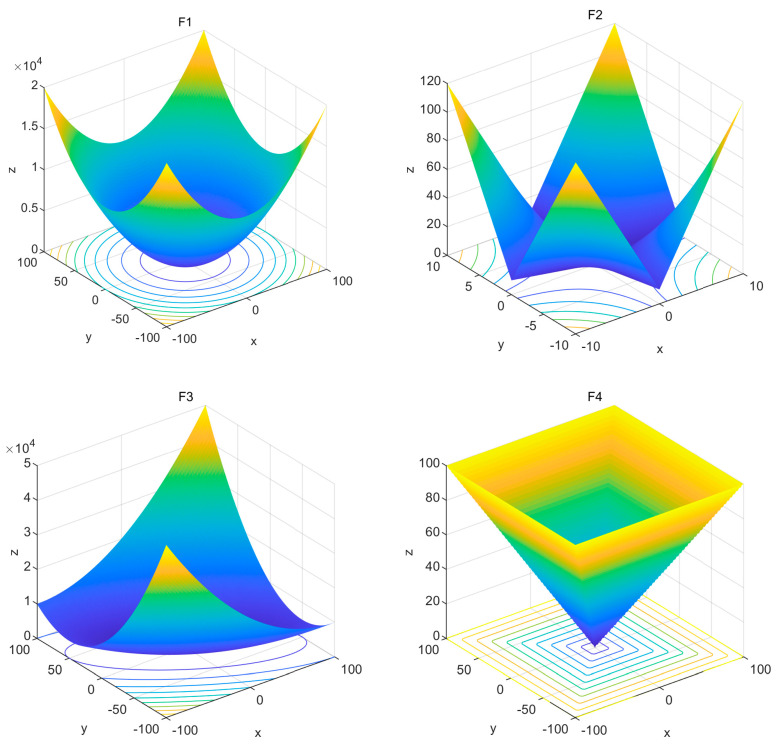

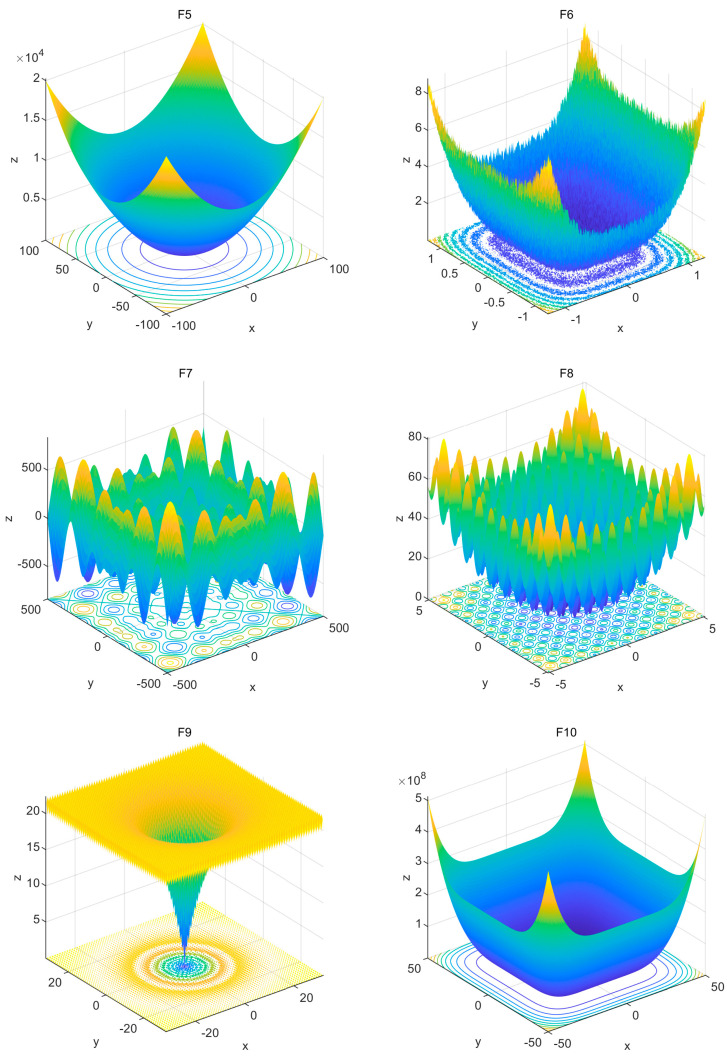
Images of test functions.

Table 1 gives the details of the test functions selected in this paper, which contain the problem dimension n, the search space S, and the theoretical optimal value $f_{min}$. The single-peak functions F1–F5 can evaluate the algorithms’ solution accuracy and convergence speed, while the multi-peak functions F6–F10 test the algorithms’ ability to avoid falling into local optima and their performance of optimization on the global space search. In order to ensure the fairness and rationality of the experiments, the population size of each algorithm is set to 30, and the maximum number of iterations is set to 1000. Moreover, in order to test the stability of the algorithms, the five algorithms are run 30 times, and the optimal value, the worst value, the median, the mean, and the standard deviation of the statistical results are taken as the indicators of the algorithms’ comprehensive performance evaluation. Figure 1 shows a two-dimensional planar display of the selected test functions F1–F10, which contain both unimodal and multimodal forms.

**Figure 2:**
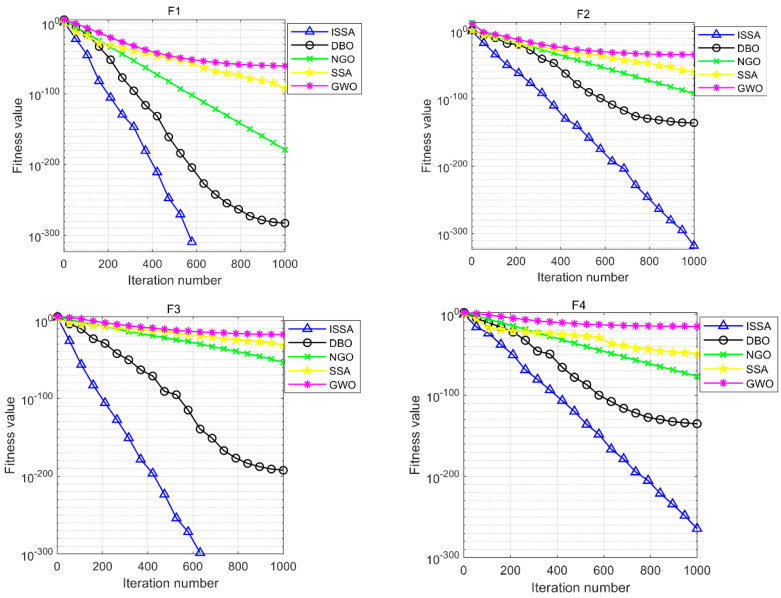

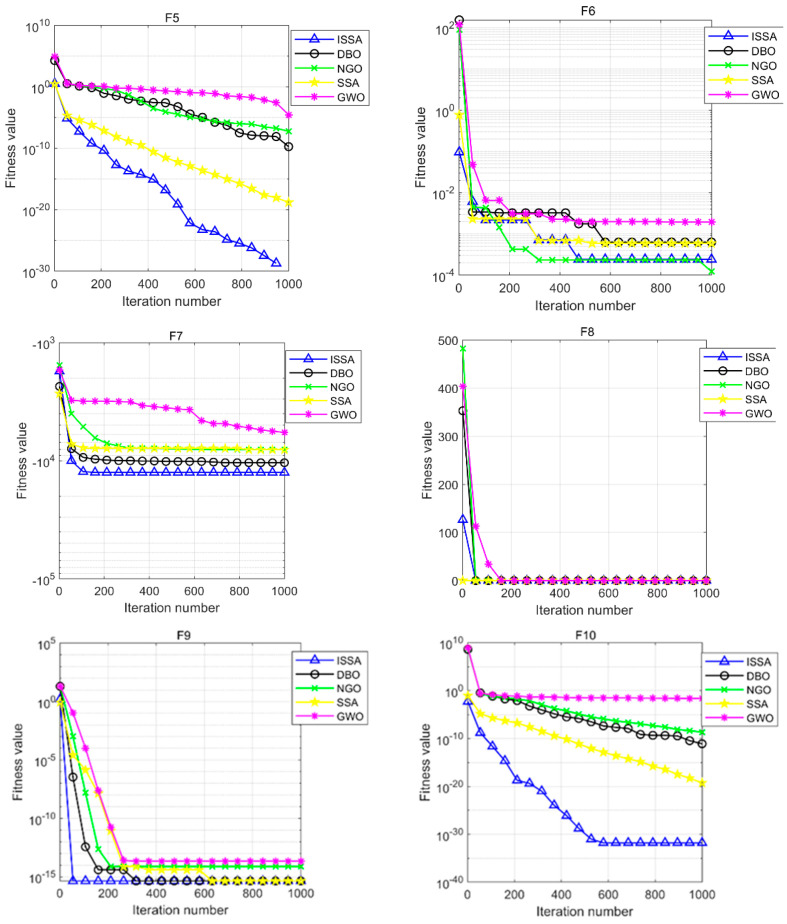
Convergence curves of five optimization algorithms on test functions.

Table 2 gives the optimization results of the ISSA and the other four algorithms when they are run independently 30 times on the 10 test functions. Figure 2 visualizes the variation of the optimization accuracy of the five algorithms when solving the 10 test functions in the search space. It is easy to see that the optimization ability of the ISSA on the single-peak functions F1–F5 is significantly better than that of the standard DBO, NGO, SSA, and GWO, and basically, it can find the optimal value. When the ISSA solves the functions F1 and F3, it can converge the standard deviation to the minimum value of 0 while maintaining a high degree of accuracy, which proves that the improved strategy has a very good effect on the improvement of the algorithm’s optimization accuracy. The experimental results obtained by the ISSA in solving functions F2 and F4 rank first by many orders of magnitude higher than the remaining four algorithms, and its stability is also very impressive. The ISSA also performs well in function F5, and when the accuracy of the remaining four algorithms is low, the ISSA still converges to the global optimum value of 0, which proves its strong optimization performance.

When solving the multi-peak test functions F6–F10, the optimization finding performance of the ISSA is still remarkable. On function F7, the ISSA can find the global optimum, while the other algorithms are prone to falling into the local optimum. The results of the ISSA on function F10 are also superior to the other algorithms, and its stability is also better than the other algorithms. On functions F6, F8, and F9, although the performance gap between the five algorithms is not very large, basically the average value of the ISSA’s optimization results in each test function is closer to the function minimum, and the standard deviation is smaller. It can be seen that the ISSA is able to control the experimental error within a smaller range and possesses more robust robustness in the case of poor optimization-seeking results.

**Figure 3:**
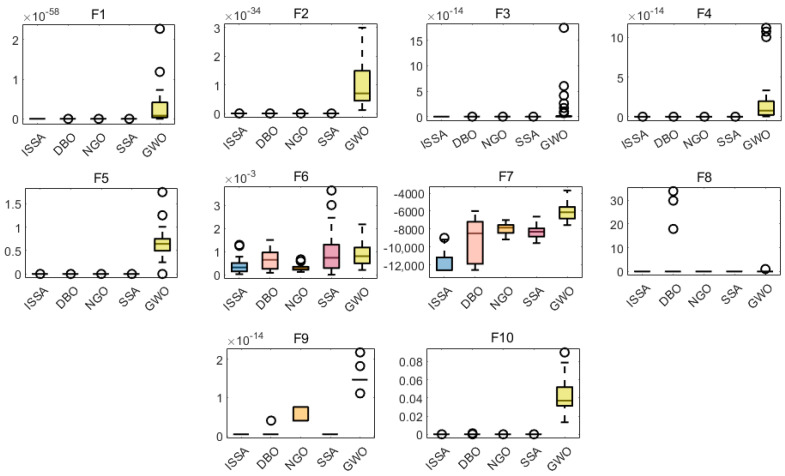
Boxplots of 10 test functions.

Figure 3 shows the boxplots of each algorithm over 30 runs. The boxplots show the central tendency, skewness, and distribution of the dataset and can highlight outliers. The figure shows that the ISSA’s results in the 10 function tests are mostly distributed in a smaller region and the outliers are sparse, and the results are generally contained between the upper and lower bounds, indicating that the overall has less distributional variability, showing its excellent solution accuracy as well as optimization stability.

The ISSA basically outperforms DBO, NGO, SSA, and GWO in terms of optimization-seeking performance for both single-peak and multi-peak test functions. The improvement does not change the complexity of the algorithm but enhances the randomness and diversity of the population in the algorithm to avoid the algorithm from falling into the local optimal solution, so as to improve the algorithm’s global search ability and optimization accuracy. After finding a potential better solution, a fine local search is carried out to improve the quality of the solution. In conclusion, the ISSA obtains more excellent performance in terms of convergence speed, solution accuracy, and robustness.

## 3. Model Construction

### 3.1. ISSA-BPNN

**Figure 4:**
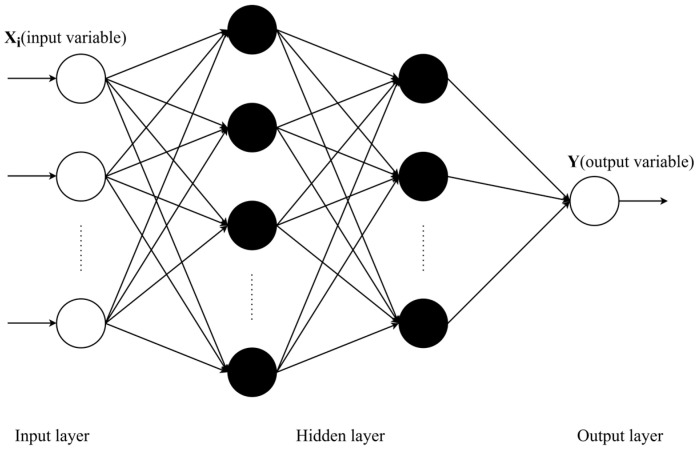
Structure of the BPNN.

ANN is a network model inspired by the structure and operation mechanism of biological nervous systems and performs information processing through a large number of interconnected artificial neurons. Among the many types of ANN, BPNN is particularly prominent and is one of the most widely used models [28]. The learning signal of BPNN is transmitted forward, the error is fed backward, the weights and thresholds are adjusted step by step so as to approach the target value, and the result is output by the transfer function. The structure of the BPNN model contains an input layer, a hidden layer, and an output layer, as shown in Figure 4.

**Figure 5:**
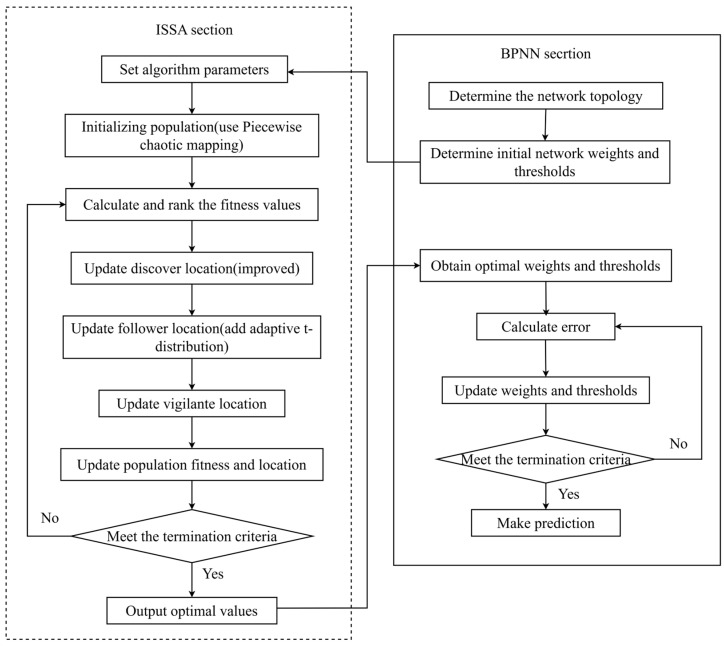
Structure of the ISSA-BPNN.

Hornik et al. [29] demonstrated that a three-layer model with one layer each of the input, hidden, and output layers is able to approximate the accuracy to any continuous function. This reflects that BPNN has the advantage of dealing with complex nonlinear relationships, so it is often introduced into concrete strength prediction. However, its prediction results are easily affected by the model structure and fitting ability, and it is prone to problems such as underfitting or overfitting, which leads to inaccurate prediction results. Therefore, in this paper, an algorithm training model is constructed by using the ISSA to search for the optimal initial weights and thresholds of BPNN and then applying them to the setup network. Compared with BPNN, the ISSA-BPNN avoids the limitations of traditional BPNN that rely on initial weight selection, thus improving the accuracy and stability of the model. The ISSA-BPNN structure is shown in Figure 5.

### 3.2. ISSA-BPNN-AdaBoost

AdaBoost is an ensemble learning model proposed by Schapire et al. in 1995 [30]. It iteratively trains a series of weak classifiers, calculates the adjustment weights, and then combines them to form a strong classifier, which results in a strong classifier with better generalization ability. AdaBoost is mainly applied to classification problems, but its principles can also be applied to regression problems, and this extension is called AdaBoost Regression [31]. In the regression task, the basic idea of AdaBoost remains the same, which is to combine multiple weak predictive models in an iterative manner to build a strong predictive model.

**Figure 6:**
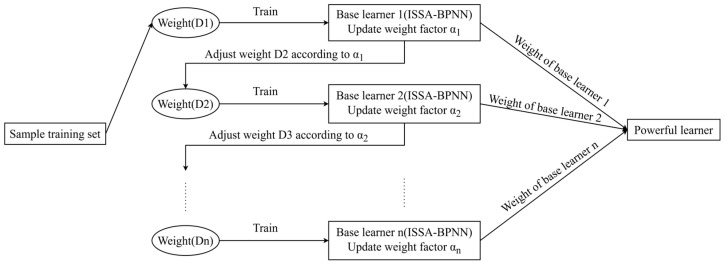
Structure of the ISSA-BPNN-AdaBoost.

In order to further improve the fitting prediction performance for concrete compressive strength, ISSA-BPNN was used as the base learner, and the AdaBoost algorithm was used to train and ensemble multiple base learners, thus constructing the ISSA-BPNN-AdaBoost concrete compressive strength prediction model. In this model, we used 10 base learners, and the contribution of each learner to the final prediction was based on its error rate. The ISSA-BPNN-AdaBoost structure is shown in Figure 6. The specific steps are as follows:

1. Preprocess the dataset by dividing it into a training set and a test set. Set the maximum number of iterations (i.e., the number of base learners). Initialize the weights of each training sample.
2. Train the current base learner based on the distribution of the weights of the current training samples using the ISSA-BPNN model as the base learner.
3. Calculate the error rate and weights of the current weak learner respectively.
4. Update the weights of each training sample.
5. Check whether the maximum iteration count is reached. If yes, stop the algorithm iteration and combine all the base learners obtained during the training process to obtain the final strong learner. (Otherwise, the process jumps back to step 2 to continue training a new base learner.)
6. Use the strong learner to train and predict the test dataset and output the final result.

### 3.3. Model Evaluation Indicators

In order to comprehensively evaluate the model developed in this paper, root mean square error (RMSE), mean absolute error (MAE), and correlation coefficient (R^2^) were used as performance metrics for model prediction [32].

1. RMSE measures the average error produced by the model in making predictions and is the square root of the mean squared error (MSE), which is the average squared difference between the actual data values and the model predictions. Typically, the lower the RMSE, the better the model. RMSE is calculated using the following formula:

(8) $$RMSE=\sqrt{\frac{1}{m}\sum_{i=1}^{m}{\left(y_{i}-\hat{y_{i}}\right)}^{2}}$$

2. MAE, like the RMSE, is an evaluation metric for measuring prediction error. This metric shows the average absolute difference between actual values and predicted results and is less sensitive to outliers than RMSE. MAE is calculated using the following formula:

(9) $$MAE=\frac{1}{m}\sum_{i=1}^{m}\left(\left|y_{i}-\hat{y_{i}}\right|\right)$$

3. R^2^, also known as the goodness of fit, compares the total variability of the model’s predicted values with the actual values and expresses the degree of fit of the model. R^2^ ranges between 0 and 1, and the closer the value is to 1, the better the fit. R^2^ is calculated using the following formula:
  (10) $$R^{2}=1-\frac{\sum_{i=1}^{m}{\left(y_{i}-\hat{y_{i}}\right)}^{2}}{\sum_{i=1}^{m}{\left(y_{i}-\bar{y_{i}}\right)}^{2}}$$
  where $y_{i}$ represents the actual value. $\hat{y_{i}}$ represents the model predicted value. $\bar{y_{i}}$ represents the mean. m represents the sample size.

## 4. Case Study Analysis

Since concrete is a composite material, water-cement ratio, aggregate size, maintenance, environmental temperature and humidity, age, concrete construction methods, and other factors will affect its compressive strength, so it is difficult to consider all the factors together. In this paper, the effect of mix on concrete strength is considered, and Dataset 1 is used to test the effectiveness of the ISSA-BPNN-AdaBoost model for concrete compressive strength prediction. Dataset 1 is from the concrete compressive strength dataset of the UCI Machine Learning Library [33]. Dataset 1 contains 1030 samples of high-strength concrete, each with eight input variables: cement, fly ash, blast furnace slag, high-efficiency water reducer, water, coarse aggregate, fine aggregate, and concrete age. In addition, there is a target output variable, which is the compressive strength of the concrete. The cement used was silicate cement (ASTM Type I). The fly ash was produced by the power plant. Water-quenched blast furnace slag powder was supplied by a local steel plant. The water was ordinary tap water. The chemical admixture was superplasticizer conforming to the ASTM C494 Type G standard. The coarse aggregate was natural gravel with a maximum particle size of 10 mm. The fine aggregate was washed natural river sand with a modulus of fineness of 3.0 [33].

### 4.1. Data Analysis and Pre-Processing

The results of the statistical analysis of Dataset 1 are shown in Table 3, which shows the mean, median, standard deviation, variance, minimum, maximum, and skewness for each variable in the dataset used. In order to reveal the degree of correlation between each type of input variable and the final output variable, nine variables were correlated using the software STATA18 as shown in Table 4. The magnitude of the coefficients indicates the degree of correlation between two variables, with larger coefficients indicating a stronger correlation and a stronger linear relationship between the two variables. It should be noted that the magnitude of the correlation coefficient, although it can reflect the strength of the linear relationship between the variables, does not indicate causality. Therefore, the significance of the correlation coefficient also needs to be determined by statistical tests to ensure that the observed correlation did not occur by chance.

From Table 4, it can be seen that the correlation coefficient of cement, fly ash, highly efficient water reducing agent, and concrete age for the compressive strength of concrete is positive, indicating that these variables are positively correlated with the compressive strength of concrete, and with the increase in the amount, the strength of concrete will be increased. The correlation coefficients of blast furnace slag, water, coarse aggregate, and fine aggregate for concrete compressive strength are negative, indicating that these variables are negatively correlated with the concrete compressive strength, and with the increase in the amount, the concrete strength will be reduced. In addition, the effect of cement admixture, highly efficient water reducing agent, water content, and age of concrete on the compressive strength of concrete is significantly greater than the other variables, which proves that these four variables have a greater effect on the compressive strength of concrete. In addition, the correlation coefficients of all variables did not reach more than 0.8, which indicates that there is no covariance problem between the variables, and it is not necessary to delete the variables.

From the dataset, it can be seen that the unit size of each variable is different, which will greatly affect the results of neural network training and prediction, which requires data normalization, so as to improve the convergence speed of the model and to avoid the impact of the differences between the features on the training of the model. In this paper, we use max-min normalization to normalize the data with the following formula:

(11) $$Y=\frac{X-X_{min}}{X_{max}-X_{min}}\;$$

where Y represents the normalization result. $X_{min}$ represents the minimum value in the sample. $X_{max}$ represents the maximum value in the sample. X is the sample value to be normalized.

### 4.2. Hyperparameter Setting

For BPNN, the setting of the hidden layer will directly affect the performance and prediction accuracy of the network. Using too few neurons in the hidden layer will result in underfitting, making the model unable to learn the data features well, thus affecting the prediction ability. On the contrary, using too many neurons will lead to overfitting and make it difficult to achieve the expected results. Therefore, choosing an appropriate number of hidden layer neurons is crucial. Determination of the number of hidden layer units in a BPNN is a complex problem with no fixed answer, so in this paper, we use the trial-and-error method to determine the number of hidden layer neurons in a single weak learner. First, the empirical formula is used to calculate the value range of the hidden layer neurons, then the number of hidden layer neurons is set in this range, and after many training repetitions, the number of hidden layer neurons with the smallest training error of BPNN is chosen. The commonly used empirical formula is as follows:

(12) $$S=\sqrt{M+N}+A\;$$

where S, M, and N represent the number of nodes in the hidden, input, and output layers, respectively. A is a constant in the interval [0,10].

**Figure 7:**
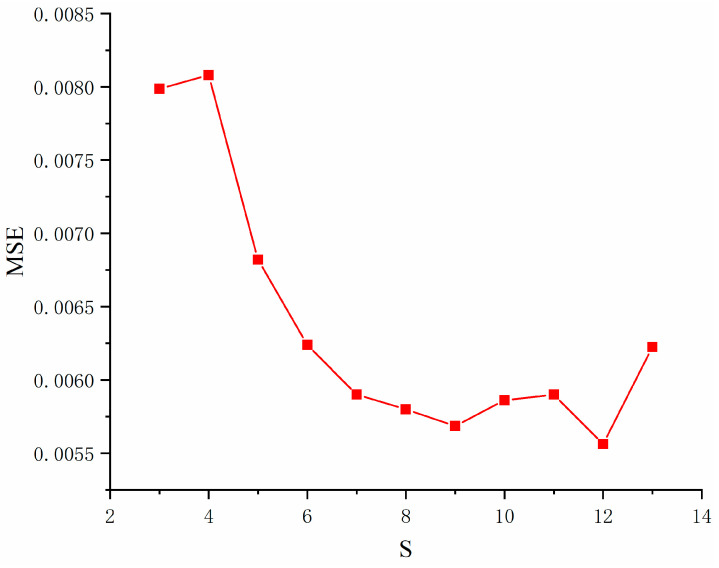
Training errors for different hidden layer neurons.

According to the empirical formula, the range of the number of hidden layer neurons is obtained as [3,13]. Different numbers of hidden layer neurons in the interval are substituted into the ISSA-BPNN model for multiple trainings, and the training errors of different hidden layer neurons are obtained as shown in Figure 7. The results show that the training error of the model is minimized when the number of neurons in the hidden layer in the BPNN is 12. Therefore, in this paper, the number of hidden layer neurons in the neural network of the weak learner is set to 12, and the three-layer structure of the neural network used consists of eight input neurons, 12 hidden layer neurons, and one output layer neuron. For determining the other hyperparameters, 10-fold cross-validation was used. All the training datasets were divided into 10 subsets of the same size, and one of the subsets was used in turn as the validation set for validation, and the remaining portion was used as the training set for training. This was repeated 10 times, and the final performance was taken as the average of the 10 experiments [34]. By combining this with a grid search, the best hyperparameter pairing can be found. The main hyperparameters are shown in Table 5.

### 4.3. Comparison with Ensemble Models

The compressive strength of concrete and the compressive strength prediction results of the ISSA-BPNN-AdaBoost model are compared and analyzed with those of several ensemble learning models, namely RF, AdaBoost, and XGBoost. RF is an ensemble learning method that constructs multiple decision trees and votes or averages their results to obtain a final prediction. XGBoost is an efficient gradient boosting algorithm that improves the performance of the model by incrementally adding prediction trees, where each weak learner is fitted against the residuals of the previous learner to gradually approximate the true value. In addition, it limits the complexity of the model by introducing regularization terms to prevent overfitting.

In order to clarify the optimal partition ratio, the dataset was partitioned and tested using three ratios of 7:3, 8:2, and 9:1 for the training set over the test set. In this study, 30 independent calculations were carried out using multiple runs, and the average statistical results are given. Table 6 gives the specific evaluation metrics of the four machine learning algorithms on the training and test sets. Although sometimes other segmentation ratios work better in the test set, overall, better training and prediction results are obtained using the 8:2 ratio segmentation model.

The 8:2 split ratio was analyzed. On the training set, the ISSA-BPNN-AdaBoost model performs the best with RMSE, MAE, and R^2^ of 3.524, 2.582, and 0.971, respectively, and the difference in the performance of the other ensemble models is not particularly large. The RMSE and MAE of the ISSA-BPNN-AdaBoost model decreased by 11.57% and 12.83%, respectively, compared to the AdaBoost model; 10.54% and 16.87%, respectively, compared to the XGBoost model; and 9.27% and 16.17%, respectively, compared to the RF model. Compared to the AdaBoost model, R^2^ increased by 2.75%; compared to the XGBoost model, it increased by 3.08%; and compared to the RF model, it increased by 4.75%. This indicates that the ISSA-BPNN-AdaBoost model can fit the training data well and has high prediction accuracy.

On the test set, the ISSA-BPNN AdaBoost model also performed well, with RMSE, MAE, and R^2^ of 3.548, 2.954, and 0.964, respectively. The RMSE and MAE of the ISSA-BPNN-AdaBoost model decreased by 25.24% and 14.38%, respectively, compared to the AdaBoost model; 30.51% and 23.95%, respectively, compared to the XGBoost model; and 34.73% and 25.59%, respectively, compared to the RF model. Compared to the AdaBoost model, R^2^ increased by 6.64%; compared to the XGBoost model, it increased by 6.64%; and compared to the RF model, it increased by 8.80%. This means that the ISSA-BPNN-AdaBoost model not only performs well on training data but also maintains good generalization ability on unseen new data, which can be used to predict the strength of concrete. It can be seen that there are conspicuous differences on the test set. Optimizing the base learner enables the ensemble model to achieve better generalization ability, reduces overfitting to the training set, and improves the predictive ability of the model.

**Figure 8:**
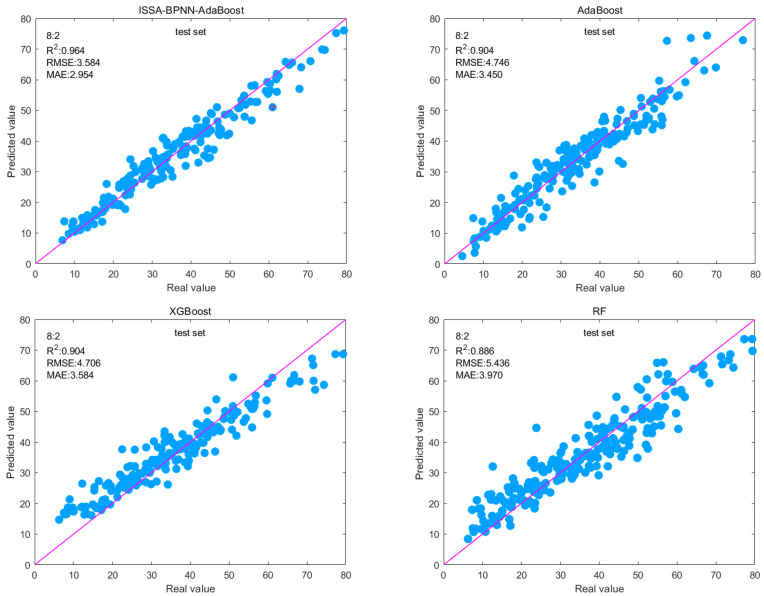
Fitted plots of predicted and actual values in the test set for each ensemble model.

The prediction results presented in Figure 8 can be used as a reference for assessing the predictive ability of the four models. The data points of the prediction results of the four models are scattered around the baseline (y = x). It can be seen that the difference between the predicted and actual values of the ISSA-BPNN-AdaBoost model is small, and the data points are basically arranged around the baseline, which indicates that its prediction results are still more accurate.

### 4.4. Comparison with Single Model

In order to test the performance of the base learner ISSA-BPNN so as to further evaluate the prediction effect of the ISSA-BPNN-AdaBoost model, five single models, namely, BPNN, SVM, convolutional neural network (CNN), extreme learning machine (ELM), and long short-term memory neural network (LSTM), were also selected to conduct the concrete strength prediction experiments with the ISSA-BPNN in this study. SVM regression, also known as support vector regression (SVR), is based on finding an optimal hyperplane that minimizes the distance between the hyperplane and the sample points. By minimizing this distance, the regression function is obtained to fit the training sample as closely as possible. CNN is a deep learning model that excels in image processing and classification tasks. However, CNN can also be used for regression problems, where convolutional layers are used to extract features from data, pooling layers are used to reduce the size of feature maps, and fully connected layers are used to integrate features and perform regression analysis. ELM is a feed-forward neural network learning algorithm whose core idea is to generate weights and biases layer by layer from the input layer to the hidden layer and then calculate the weights of the output layer directly by least squares or other optimization methods. This reduces the need for iterative computation of the hidden layer weights, thus increasing the training speed. Finally, LSTM is a special type of recurrent neural network that introduces input gates, forget gates, and output gates to control the information flow, thereby solving the problems of gradient vanishing and exploding that may occur in traditional RNNs.

In addition, three different data splitting ratios of 7:3, 8:2, and 9:1 were equally selected for each model. The computational results of each model are shown in Table 7. It can be seen that in general, the use of the 8:2 ratio segmentation model can get better training and prediction results, while the use of the 9:1 ratio will have a certain overfitting phenomenon, resulting in a decrease in prediction accuracy instead.

**Figure 9:**
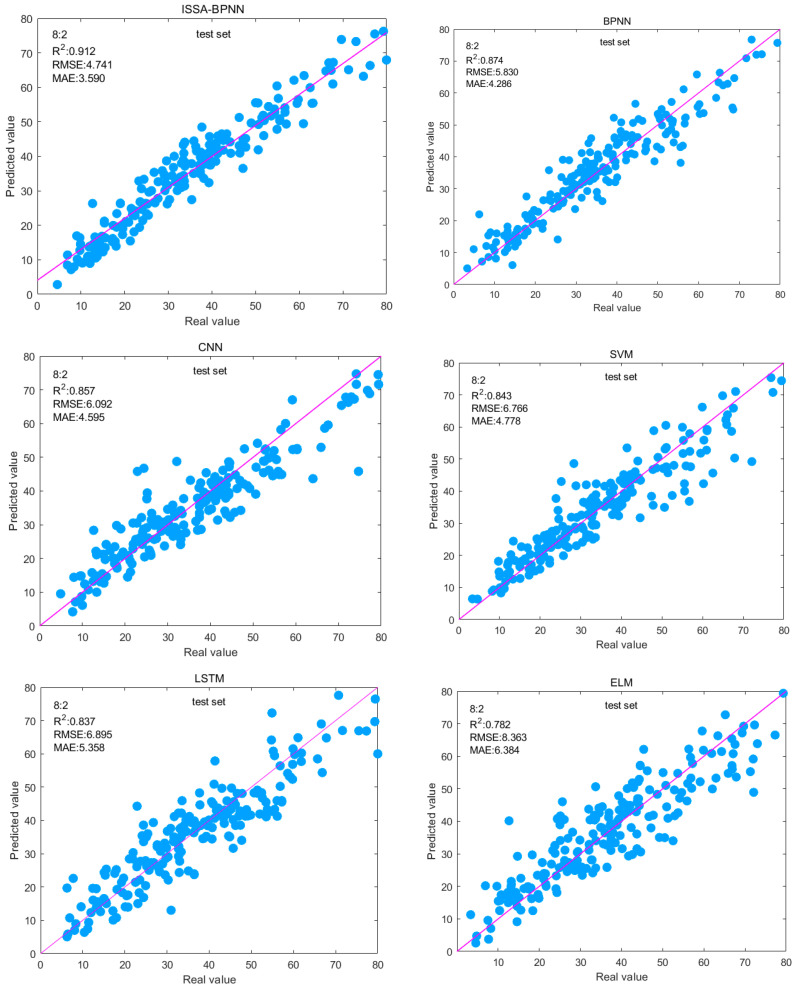
Fitted plots of predicted and actual values in the test set for each single model.

The 8:2 split ratio was analyzed, and the six models’ R^2^ values were in descending order of ISSA-BPNN > BPNN > CNN > SVM > LSTM > ELM, and the ISSA-BPNN model obtained the best result. Compared with the base BPNN model, the RMSE and MAE of the test set of the ISSA-BPNN model decreased by 18.68% and 16.24%, respectively, and the R^2^ improved by 4.35%. This indicates that ISSA improves the prediction accuracy and stability of the BPNN model by optimizing the initial weights and thresholds of the BPNN. Figure 9 shows the fitting effect between the predicted and actual values of the test set of each model, from which it can be intuitively seen that the fitting effect of the ISSA-BPNN model is the best among the other models, and the sample points are relatively more concentrated on the base line. The ISSA-BPNN has a fast convergence speed and strong global optimization ability and can accurately predict the concrete compressive strength. Then the ISSA-BPNN-AdaBoost model using it as a base learner will have much better performance. Comparing the evaluation metrics of the ISSA-BPNN-AdaBoost model with the above models, it can be seen that the ensemble learning model outperforms all these single models and the ISSA-BPNN model in predicted effect, and the R^2^ is also improved by 5.70% compared to the ISSA-BPNN model.

In summary, whether it is the training set or the test set, among the above machine learning regression algorithms, the three evaluation indexes of the ISSA-BPNN-AdaBoost model are optimal, indicating that the data predicted by the ISSA-BPNN-AdaBoost algorithm model fit well with the real data, and the accuracy and reliability of the prediction results are better than those of other models.

## 5. Model Performance Verification

In order to further demonstrate the feasibility of the ISSA-BPNN-AdaBoost model when applied in practice and to test the generalization ability of the model, a new dataset was chosen for testing. Dataset 2 is from Chopra et al. [35] and lists 76 concrete mixes and their compressive strengths, of which 49 are without fly ash, 27 are with fly ash, and neither blast furnace slag nor high-efficiency water reducer are included. Dataset 2 uses ordinary silicate cement (OPC) of grade 43 with a specific gravity of 3.12. The aggregate has a specific gravity of 2.54 and a fineness modulus of 2.09. The sand conforms to Zone III standards. The coarse aggregate used here consists of two sizes, 20 mm and 10 mm, with specific gravity values of 2.61 and 2.63, respectively, mixed in different proportions [35]. Each sample in Dataset 2 has five input variables: cement, fly ash, water, coarse aggregate, and fine aggregate. In addition, there is one target output variable, the compressive strength of the concrete. Again, the dataset was analyzed statistically as shown in Table 8, which shows the mean, median, standard deviation, variance, minimum, maximum, and skewness for each variable in the dataset used. The variables were also correlated using STATA software, as shown in Table 9. It can be seen that water, coarse aggregate, fine aggregate, and fly ash are likewise negatively correlated with strength, and cement is positively correlated with strength, which is the same as the results shown in Dataset 1.

This time, three models, BPNN, ISSA-BPNN, and ISSA-BPNN-AdaBoost, were used for testing with a train-test split ratio of 8:2. The specific evaluation metrics of the three models on the training and testing sets of Dataset 2 are given in Table 10. From the results of the evaluation metrics, it can be seen that the ISSA-BPNN-AdaBoost model has the best prediction effect on Dataset 2. The values of the RMSE and MAE evaluation metrics are smaller on both the training set and the test set, which indicates that the average error between the predicted value and the real value is smaller, the model fits better, and the prediction accuracy is higher. Finally, the results of the R^2^ evaluation metrics are 0.982 and 0.969, showing high accuracy on both the training set and the test set, indicating that the model has a good fitting ability and a strong prediction ability. In addition, after several experiments, the prediction performance of the BPNN model is found to be very unstable, which may be due to the small dataset, and the difference in the training set selected each time will greatly affect the prediction accuracy of the BPNN model. In contrast, the ISSA-BPNN-AdaBoost model has a more stable performance in each experiment, which indicates that the model can also cope with the prediction needs when the data are small.

**Figure 10:**
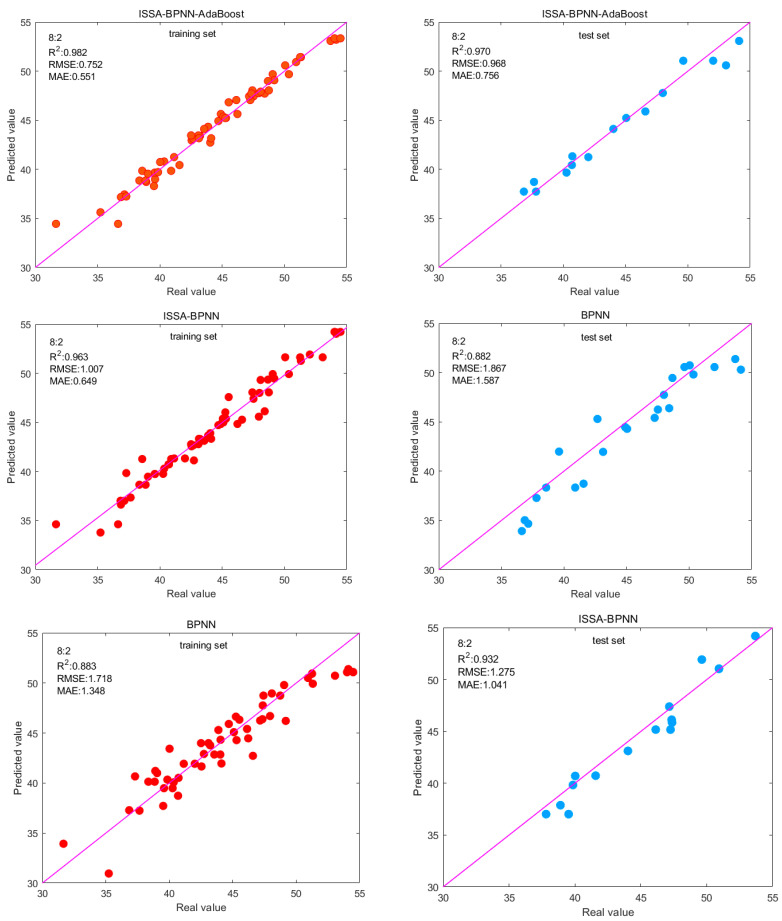
Fitted plots of predicted and actual values in the training set and test set for each model.

Figure 10 illustrates the prediction results of the three models. The data points of the prediction results of the three models are scattered around the baseline. It can be seen that the ISSA-BPNN-AdaBoost model has high prediction accuracy, and the points are closely surrounding the baseline with a strong prediction ability.

## 6. Conclusions

In order to improve the accuracy of the model in predicting the compressive strength of concrete, the base learner of the ensemble learning model is optimized in this paper. The SSA is improved by adding strategies such as piecewise chaotic mapping and adaptive t-distribution variation and improving the position update formula in the search phase. Subsequently, ISSA was used to search for the optimal initial weights and thresholds of BPNN, which was then used as the base learner of AdaBoost to establish the ISSA-BPNN-AdaBoost concrete compressive strength prediction model. The ISSA-BPNN-AdaBoost model was compared and analyzed with other models in two different datasets, and the following conclusions were obtained:

1. Instead of increasing its complexity, the improvements to SSA improve its poor initial population quality, poor ability to jump out of local optima, and dimensionality shrinkage. On the 10 general benchmark test functions, ISSA achieves better performance, and ISSA’s optimization performance is basically better than the four basic intelligent optimization algorithms, DBO, NGO, SSA, and GWO, in both single-peak and multi-peak test functions, and it has better convergence speed and optimization accuracy.
2. In Dataset 1, the ISSA-BPNN-AdaBoost model test set achieves a goodness-of-fit of 0.964. Such a goodness-of-fit can satisfy the requirements of actual prediction accuracy. Compared with the compared ensemble models, the R^2^ of the ISSA-BPNN-AdaBoost model test set is 6.64% better than the AdaBoost model, 6.64% better than the XGBoost model, and 8.80% better than the RF model. The R^2^ of the ISSA-BPNN-AdaBoost model test set is also improved by more than 10% compared with other comparative single models. The RMSE, MAE, and R^2^ of the ISSA-BPNN-AdaBoost model are optimal in the training set and the test set, which indicates that its prediction data have the best fit with the real data, and the accuracy and reliability of its predictions are better than those of the other models.
3. The generalized prediction ability of the ISSA-BPNN-AdaBoost model is well validated in Dataset 2. The model achieved an R^2^ value of 0.970 on the test set, implying that the model was able to account for most of the data variability, a result that suggests that the model has a very high prediction accuracy. In addition, the RMSE and MAE of the model are both very low, further confirming the model’s excellent performance in generalization ability. On small datasets, by adopting an integrated strategy, the model can avoid overfitting the training set, which demonstrates its ability to generalize to new data while remaining sensitive to variable relationships.

In summary, the ISSA-BPNN-AdaBoost model proposed in this study can predict the concrete compressive strength more accurately, has good generalization ability, and can provide a reference for practical engineering. The study in this paper mainly focuses on the prediction of the static compressive strength of concrete and does not involve dynamic strength. In addition, hyperparameter optimization is the focus of developing excellent models, and the use of more effective hyperparameter optimization strategies such as Optuna [36] is an important research direction that needs to be continued in future studies.

## Figures and Tables

**Table 1:** Test functions.

	Test Function	n	S	$f_{min}$
F1	$f\left(x\right)=\sum_{i=1}^{n}x_{i}^{2}$	30	${\left[-100,100\right]}^{n}$	0
F2	$f\left(x\right)=\sum_{i=1}^{n}\left|x_{i}\right|+\prod_{i=1}^{n}\left|x_{i}\right|$	30	${\left[-10,10\right]}^{n}$	0
F3	$f\left(x\right)=\sum_{i=1}^{n}{\left(\sum_{j=1}^{i}x_{i}\right)}^{2}$	30	${\left[-100,100\right]}^{n}$	0
F4	$f\left(x\right)={max}_{i}\left\{\left|x_{i}\right|,1\leq i\leq n\right\}$	30	${\left[-100,100\right]}^{n}$	0
F5	$f\left(x\right)=\sum_{i=1}^{n}{\left(\left|x_{i}+0.5\right|\right)}^{2}$	30	${\left[-100,100\right]}^{n}$	0
F6	$f\left(x\right)=\sum_{i=1}^{n}ix_{i}^{4}+random\left[0,1\right)$	30	${\left[-1.28,1.28\right]}^{n}$	0
F7	$f\left(x\right)=\sum_{i=1}^{n}-x_{i}\sin\left(\sqrt{\left|x\right|}\right)$	30	${\left[-500,500\right]}^{n}$	−12,569.5
F8	$f\left(x\right)=\sum_{i=1}^{n}\left[x_{i}^{2}-10\cos\left(2\pi x_{i}\right)+10\right]$	30	${\left[-5.12,5.12\right]}^{n}$	0
F9	$f\left(x\right)=-20exp\left(-0.2\sqrt{1/n\sum_{i=1}^{n}x_{i}^{2}}\right)-\exp\left(1/n\sum_{i=1}^{n}\cos2\pi x_{i}\right)$ + 20 + e	30	${\left[-32,32\right]}^{n}$	0
F10	$\begin{array}{c} f\left(x\right)=\pi /n10\sin^{2}\left(\pi y_{i}\right)+\sum_{i=1}^{n-1}{\left(y_{i}-1\right)}^{2}\left[1+10\sin^{2}\left(\pi y_{i+1}\right)\right]+{\left(y_{n}-1\right)}^{2}+\\ \sum_{i+1}^{n}u\left(x_{i},10,1000,4\right),\;y_{i}=1+1/4\left(x_{i}+1\right),\;u\left(x_{i},a,k,m\right)=\left\{\begin{array}{c} k{\left(x_{i}-a\right)}^{m}{\;\;\;\;\;x}_{i}>a\\ 0\;\;\;\;\;\;\;\;\;\;-a\leq x_{i}\leq a\\ k{\left({-x}_{i}-a\right)}^{m}\;\;\;x_{i}<-a \end{array}\right. \end{array}$	30	${\left[-50,50\right]}^{n}$	0

**Table 2:** Comparison among optimal results of test functions.

		Optimal	Worst	Median	Average	SD
F1	ISSA	0	0	0	0	0
	DBO	0	1.81 × 10^−218^	2.61 × 10^−276^	6.04 × 10^−220^	0
	NGO	5.71 × 10^−183^	6.59 × 10^−178^	1.91 × 10^−180^	3.71 × 10^−179^	0
	SSA	0	1.44 × 10^−81^	9.68 × 10^−99^	4.81 × 10^−83^	2.63 × 10^−82^
	GWO	8.14 × 10^−62^	2.27 × 10^−58^	7.99 × 10^−60^	2.77 × 10^−59^	4.68 × 10^−59^
F2	ISSA	0	1.12 × 10^−268^	1.91 × 10^−300^	3.73 × 10^−270^	0
	DBO	1.66 × 10^−152^	4.32 × 10^−118^	1.53 × 10^−139^	1.44 × 10^−119^	7.88 × 10^−119^
	NGO	7.76 × 10^−94^	1.80 × 10^−91^	6.56 × 10^−93^	1.60 × 10^−92^	3.55 × 10^−92^
	SSA	0	1.04 × 10^−39^	8.60 × 10^−49^	4.28 × 10^−41^	1.92 × 10^−40^
	GWO	1.17 × 10^−35^	2.99 × 10^−34^	6.98 × 10^−35^	9.87 × 10^−35^	7.37 × 10^−35^
F3	ISSA	0	0	0	0	0
	DBO	1.99 × 10^−257^	2.17 × 10^−116^	1.53 × 10^−209^	7.24 × 10^−118^	3.97 × 10^−117^
	NGO	8.29 × 10^−58^	8.07 × 10^−46^	9.97 × 10^−54^	2.70 × 10^−47^	1.47 × 10^−46^
	SSA	0	2.51 × 10^−32^	8.55 × 10^−50^	8.37 × 10^−34^	4.58 × 10^−33^
	GWO	4.27 × 10^−20^	1.75 × 10^−13^	2.03 × 10^−16^	1.14 × 10^−14^	3.38 × 10^−14^
F4	ISSA	8.38 × 10^−308^	9.44 × 10^−267^	4.93 × 10^−281^	3.96 × 10^−268^	0
	DBO	3.89 × 10^−157^	7.15 × 10^−104^	1.07 × 10^−124^	2.38 × 10^−105^	1.31 × 10^−104^
	NGO	5.74 × 10^−78^	1.33 × 10^−75^	7.11 × 10^−77^	1.99 × 10^−76^	3.01 × 10^−76^
	SSA	5.49 × 10^−130^	1.55 × 10^−38^	1.07 × 10^−50^	9.14 × 10^−40^	3.44 × 10^−39^
	GWO	2.80 × 10^−16^	1.12 × 10^−13^	7.58 × 10^−15^	1.91 × 10^−14^	3.09 × 10^−14^
F5	ISSA	0	1.34 × 10^−25^	2.87 × 10^−31^	5.04 × 10^−27^	2.44 × 10^−26^
	DBO	4.25 × 10^−11^	2.30 × 10^−6^	2.47 × 10^−9^	1.25 × 10^−7^	4.34 × 10^−7^
	NGO	7.12 × 10^−9^	2.46 × 10^−7^	4.14 × 10^−8^	6.55 × 10^−8^	6.50 × 10^−8^
	SSA	2.76 × 10^−24^	9.21 × 10^−18^	1.90 × 10^−20^	7.07 × 10^−19^	2.01 × 10^−18^
	GWO	1.43 × 10^−5^	1.75	6.42 × 10^−1^	6.58 × 10^−1^	3.42 × 10^−1^
F6	ISSA	3.35 × 10^−5^	1.30 × 10^−3^	3.21 × 10^−4^	3.98 × 10^−4^	3.13 × 10^−4^
	DBO	9.02 × 10^−5^	1.51 × 10^−3^	6.54 × 10^−4^	6.70 × 10^−4^	4.25 × 10^−4^
	NGO	1.27 × 10^−5^	6.71 × 10^−4^	2.67 × 10^−4^	3.02 × 10^−4^	1.29 × 10^−4^
	SSA	9.76 × 10^−6^	3.65 × 10^−3^	7.41 × 10^−4^	1.00 × 10^−3^	9.39 × 10^−4^
	GWO	2.11 × 10^−4^	2.19 × 10^−3^	8.12 × 10^−4^	9.05 × 10^−4^	5.41 × 10^−4^
F7	ISSA	−12,569.49	−8974.77	−12,569.49	−11,794.43	1146.83
	DBO	−12,550.31	−5996.56	−8472.92	−9252.01	2303.60
	NGO	−9143.78	−6988.91	−7837.66	−7958.94	546.14
	SSA	−9558.42	−6607.98	−8286.62	−8310.38	666.62
	GWO	−7555.06	−3700.65	−612.65	−6063.92	858.72
F8	ISSA	0	0	0	0	0
	DBO	0	33.83	0	2.72	8.58
	NGO	0	0	0	0	0
	SSA	0	0	0	0	0
	GWO	0	1.01	0	3.34 × 10^−2^	1.83 × 10^−1^
F9	ISSA	4.44 × 10^−16^	4.44 × 10^−16^	4.44 × 10^−16^	4.44 × 10^−16^	0
	DBO	4.44 × 10^−16^	4.00 × 10^−15^	4.44 × 10^−16^	6.81 × 10^−16^	9.01 × 10^−16^
	NGO	4.00 × 10^−15^	7.55 × 10^−15^	7.55 × 10^−15^	5.89 × 10^−15^	1.80 × 10^−15^
	SSA	4.44 × 10^−16^	4.44 × 10^−16^	4.44 × 10^−16^	4.44 × 10^−16^	0
	GWO	1.11 × 10^−14^	2.18 × 10^−14^	1.47 × 10^−14^	1.60 × 10^−14^	2.87 × 10^−15^
F10	ISSA	1.57 × 10^−32^	3.63 × 10^−32^	1.70 × 10^−32^	1.81 × 10^−32^	4.20 × 10^−33^
	DBO	1.04 × 10^−13^	1.06 × 10^−3^	4.42 × 10^−11^	6.52 × 10^−5^	2.27 × 10^−4^
	NGO	4.83 × 10^−10^	1.75 × 10^−8^	3.38 × 10^−9^	4.83 × 10^−9^	4.32 × 10^−9^
	SSA	5.30 × 10^−24^	6.82 × 10^−18^	2.52 × 10^−21^	2.51 × 10^−20^	1.24 × 10^−18^
	GWO	1.32 × 10^−2^	9.00 × 10^−2^	3.70 × 10^−2^	4.16 × 10^−2^	1.96 × 10^−2^

SD: standard deviation

**Table 3:** Descriptive statistics for factors in Dataset 1.

Parameters	Mean	Median	SD	Variance	Min	Max	Skewness
Water (kg/m^3^)	181.57	185.00	21.34	455.56	121.80	247.00	0.07
Cement (kg/m^3^)	281.17	272.90	104.46	10,910.98	102	540	0.51
Fine aggregate (kg/m^3^)	773.58	779.50	80.14	6421.95	594.00	992.60	−0.25
Coarse aggregate (kg/m^3^)	972.92	968.00	77.72	6039.81	801.00	1145.00	−0.04
Fly ash (kg/m^3^)	54.19	0.00	63.97	4091.64	0.00	200.10	0.54
Slag(kg/m^3^)	73.90	22.00	86.24	7436.90	0.00	359.40	0.80
Superplastic (kg/m^3^)	6.20	6.40	5.97	35.65	0.00	32.20	0.91
Age (days)	45.66	28.00	63.14	3986.56	1.00	365.00	3.26
Strength (MPa)	35.82	34.45	16.70	278.81	2.33	82.60	0.42

SD: standard deviation; Max: maximum; Min: minimum.

**Table 4:** Correlation analysis of Dataset 1.

	Cement	Slag	Fly Ash	Water	Superplasticizer	Coarse Aggregate	Fine Aggregate	Age	Strength
CEMENT	1.000								
SLAG	−0.275 ***	1.000							
FLY ASH	−0.397 ***	−0.324 ***	1.000						
WATER	−0.082 ***	0.107 ***	−0.257 ***	1.000					
SUPERPLASTICIZER	0.093 ***	0.043	0.377 ***	−0.657 ***	1.000				
COARSE AGGREGATE	−0.109 ***	−0.284 ***	−0.010	−0.182 ***	−0.266 ***	1.000			
FINE AGGREGATE	−0.223 ***	−0.282 ***	0.079 ***	−0.451 ***	0.223 ***	−0.179 ***	1.000		
AGE	0.082 ***	−0.044	−0.154 ***	0.278 ***	−0.193 ***	−0.003	−0.156 ***	1.000	
STRENGTH	0.498 ***	0.135 ***	−0.106 ***	−0.290 ***	0.366 ***	−0.165 ***	−0.167 ***	0.329 ***	1.000

Three asterisks (***) represent a significance level of 0.1%.

**Table 5:** Hyperparameters of the neural network.

Hyperparameter Name	Hyperparameter Value
Learning rate	0.01
Epochs	100
Max fail	6
Activation function	ReLU
Optimization algorithm	trainlm
Batch size	64

**Table 6:** Evaluation metrics results for each ensemble model.

	Ratio	Training Set			Test Set		
		RMSE	MAE	R^2^	RMSE	MAE	R^2^
ISSA-BPNN- AdaBoost	7:3	3.634	2.785	0.957	4.565	3.213	0.945
	8:2	3.524	2.582	0.971	3.548	2.954	0.964
	9:1	3.855	2.767	0.953	4.675	3.332	0.937
AdaBoost	7:3	4.332	3.238	0.932	5.196	4.003	0.906
	8:2	3.985	2.962	0.945	4.746	3.450	0.904
	9:1	4.399	3.293	0.932	4.475	3.391	0.915
XGBoost	7:3	4.096	3.095	0.941	5.868	4.606	0.895
	8:2	3.939	3.106	0.942	4.706	3.584	0.904
	9:1	4.286	3.306	0.934	6.075	4.726	0.867
RF	7:3	4.044	3.115	0.923	6.198	4.673	0.871
	8:2	3.884	3.080	0.927	5.436	3.970	0.886
	9:1	3.827	2.830	0.939	5.472	3.857	0.869

**Table 7:** Evaluation indicator results for each single model.

	Ratio	Training Set			Test Set		
		RMSE	MAE	R^2^	RMSE	MAE	R^2^
ISSA-BPNN	7:3	4.916	3.475	0.912	6.223	4.810	0.886
	8:2	5.015	3.787	0.921	4.741	3.590	0.912
	9:1	5.876	3.564	0.923	5.985	4.541	0.895
BPNN	7:3	6.083	4.406	0.864	6.623	5.010	0.845
	8:2	5.523	4.220	0.891	5.830	4.286	0.874
	9:1	5.294	3.922	0.898	5.938	4.654	0.887
SVM	7:3	5.619	3.939	0.885	6.635	4.627	0.848
	8:2	5.602	3.895	0.886	6.766	4.778	0.843
	9:1	5.331	3.739	0.898	8.655	5.639	0.726
CNN	7:3	5.933	4.531	0.878	6.530	4.865	0.834
	8:2	5.360	4.089	0.899	6.092	4.595	0.857
	9:1	5.655	4.225	0.885	6.585	5.193	0.854
ELM	7:3	7.324	5.698	0.816	7.197	5.504	0.792
	8:2	7.129	5.497	0.810	8.363	6.384	0.782
	9:1	7.231	5.554	0.815	7.719	5.830	0.753
LSTM	7:3	7.315	5.694	0.813	7.139	5.434	0.805
	8:2	6.400	4.969	0.851	6.895	5.358	0.837
	9:1	7.105	5.595	0.821	7.035	5.724	0.789

**Table 8:** Descriptive statistics for factors in Dataset 2.

Parameters	Mean	Median	SD	Variance	Min	Max	Skewness
Water (kg/m^3^)	202.81	199.75	12.82	164.37	178.50	229.5	0.11
Cement (kg/m^3^)	433.88	450.00	34.81	1211.73	350.00	475	−0.67
Fine aggregate (kg/m^3^)	524.31	526.50	69.38	4813.32	175.95	641.75	−1.66
Coarse aggregate (kg/m^3^)	1050.88	1096.50	134.51	18,094.07	798.00	1253.75	−0.57
Fly ash (kg/m^3^)	24.03	0.00	32.64	1065.44	0.00	71.25	0.63
Strength (MPa)	44.37	44.08	5.21	27.17	31.66	54.49	0.07

SD: standard deviation; Max: maximum; Min: minimum.

**Table 9:** Correlation analysis of Dataset 2.

	Water	Cement	Fine Aggregate	Coarse Aggregate	Fly Ash	Strength
WATER	1.000					
CEMENT	0.503 ***	1.000				
FINE AGGREGATE	0.510 ***	0.008	1.000			
COARSE AGGREGATE	−0.289 **	−0.351 ***	0.193 *	1.000		
FLY ASH	0.089	0.386 ***	−0.027	−0.140	1.000	
STRENGTH	−0.173	0.505 ***	−0.317 ***	−0.073	−0.361 ***	1.000

Three asterisks (***) represent a significance level of 0.1%; two asterisks (**) represent a significance level of 1%; one asterisk (*) represents a significance level of 5%.

**Table 10:** Evaluation indicator results for each single model.

	Training Set			Test Set		
	RMSE	MAE	R^2^	RMSE	MAE	R^2^
ISSA-BPNN-AdaBoost	0.752	0.551	0.982	0.968	0.756	0.970
ISSA-BPNN	1.007	0.649	0.963	1.275	1.041	0.932
BPNN	1.718	1.348	0.883	1.867	1.587	0.882

## Data Availability

The two datasets used have declared source articles in the text. Some or all data, models, or code that support the findings of this study are available from the corresponding author upon reasonable request.
